# Lipid Nanoparticle‐Mediated Delivery of CRISPR‐Cas9 Against Rubicon Ameliorates NAFLD by Modulating CD36 Along with Glycerophospholipid Metabolism

**DOI:** 10.1002/advs.202400493

**Published:** 2024-06-18

**Authors:** Yu Bai, Yanyang Nan, Tao Wu, An Zhu, Xinlei Xie, Yun Sun, Yong Deng, Zihan Dou, Xiaozhi Hu, Rongrui Zhou, Shuwen Xu, Yuanzhen Zhang, Jiajun Fan, Dianwen Ju

**Affiliations:** ^1^ Department of Biological Medicines & Shanghai Engineering Research Center of Immunotherapeutics Fudan University School of Pharmacy Shanghai P. R. China; ^2^ Department of Research and Development Shanghai Proton and Heavy Ion Center Fudan University Cancer Hospital Shanghai 201321 P. R. China; ^3^ Fudan Zhangjiang Institute Shanghai 201203 P. R. China; ^4^ Shanghai Hailu Biological Technology Co., Ltd. Shanghai 201200 P. R. China

**Keywords:** CD36, CRISPR‐Cas9, glycerophospholipid metabolism, LNP, NAFLD, Rubicon

## Abstract

Non‐alcoholic fatty liver disease (NAFLD) is a prominent cause of various chronic metabolic hepatic diseases with limited therapeutics. Rubicon, an essential regulator in lysosomal degradation, is reported to exacerbate hepatic steatosis in NAFLD mice and patients, indicating its probability of being a therapeutic target for NAFLD treatment. In this study, the therapeutic potential of Rubicon blockage is investigated. Lipid nanoparticles carrying Rubicon‐specific CRISPR‐Cas9 components exhibited liver accumulation, cell internalization, and Rubicon knockdown. A single administration of the nanoparticles results in attenuated lipid deposition and hepatic steatosis, with lower circulating lipid levels and decreased adipocyte size in NAFLD mice. Furthermore, the increase of phosphatidylcholine and phosphatidylethanolamine levels can be observed in the NAFLD mice livers after Rubicon silencing, along with regulatory effects on metabolism‐related genes such as CD36, Gpcpd1, Chka, and Lpin2. The results indicate that knockdown of Rubicon improves glycerophospholipid metabolism and thereby ameliorates the NAFLD progression, which provides a potential strategy for NAFLD therapy via the restoration of Rubicon.

## Graphical abstract

A single dose of the lipid nanoparticles containing CRISPR‐Cas9 components against Rubicon (sgRubicon‐LNP) alleviates hepatic steatosis, diminishes liver lipid accumulation, downregulates circulating lipid levels, and decreases adipocyte size in NAFLD mice. The silence of Rubicon upregulates the levels of phosphatidylcholine and phosphatidylethanolamine and reduces CD36 expression, indicating the therapeutic potential of Rubicon blockage for NAFLD.

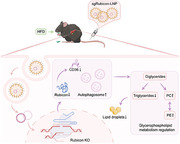

## 1 Introduction

As the most prevalent chronic hepatic disease, non‐alcoholic fatty liver disease (NAFLD) afflicts around a quarter of the global population, yet treatment options remain limited, presenting major public health challenges.^[^ ^1^, ^2^, ^3^ ^]^ At present, the primary clinical intervention for managing NAFLD is through lifestyle modification, such as exercise and weight reduction. However, the therapeutic efficiency of these interventions is often inadequate, leading to the progression of the disease to more severe forms, including steatohepatitis, cirrhosis, and relevant metabolic co‐morbidities, ultimately resulting in a high mortality rate.^[^ ^4^, ^5^, ^6^ ^]^ To address the clinical needs of NAFLD treatment, it is crucial to explore novel therapeutic strategies.

The multi‐hit theory underscores the pivotal role of metabolic dyslipidemia in the early stages of NAFLD,^[^ ^7^, ^8^, ^9^ ^]^ although the biological pathogenesis is yet to be fully deciphered. The uncontrolled accumulation of lipid droplets primarily stems from an increased influx and impaired breakdown of intrahepatic triglycerides.^[^ ^10^, ^11^ ^]^ This lipid buildup in the liver subsequently triggers endoplasmic reticulum stress, organelle dysfunction, cellular damage, and chronic inflammation.^[^ ^12^, ^13^ ^]^ The interventions targeting abnormal lipid accumulation seem to be a manageable therapeutic approach.^[^ ^14^ ^]^ Consequently, the molecules associated with lipid accumulation have emerged as attractive targets for NAFLD therapy.

Rubicon/RUBCN is identified as a RUN domain‐containing Beclin‐1‐interacting protein with various functions in fundamental biological processes such as autophagy, phagocytosis, endocytosis, microbial infection, innate immune response, and aging process.^[^ ^15^, ^16^, ^17^ ^]^ Growing evidence showed that Rubicon might be a pivotal component in lipid metabolic regulation. An age‐dependent decline in Rubicon expression in adipocytes has been shown to cause fat atrophy, indicating its essential role in adipogenesis pathway.^[^ ^18^, ^19^ ^]^ Encouragingly, a recent study showed that Rubicon was upregulated during NAFLD progression and hepatocyte‐specific Rubicon deficiency mice displayed slighter liver steatosis and endoplasmic reticulum.^[^ ^20^ ^]^ Furthermore, suppression of the methyltransferase like 3 (METTL3) led to reduced Rubicon expression, facilitating lipid droplet clearance.^[^ ^21^ ^]^ These findings suggest Rubicon's critical role in lipid metabolism regulation, however, its viability as a NAFLD therapeutic target remains uncertain.

In the present study, we developed a single‐dose‐usage lipid nanoparticle (LNP) for NAFLD treatment by delivering CRISPR‐Cas9 components against Rubicon (sgRubicon‐LNP). The bio‐behavior and therapeutic efficacy of the nanoparticles in NAFLD mice were investigated in vitro and in vivo. To elucidate the underlying mechanisms, the changes in glycerophospholipid metabolism and regulatory molecules such as CD36 were evaluated following sgRubicon‐LNP administration. Our results suggested that Rubicon blockage was a promising strategy to attenuate the progression of NAFLD.

## 2 Results

### 2.1 Synthesis and Characterization of sgRubicon‐LNP

**Figure 1:**
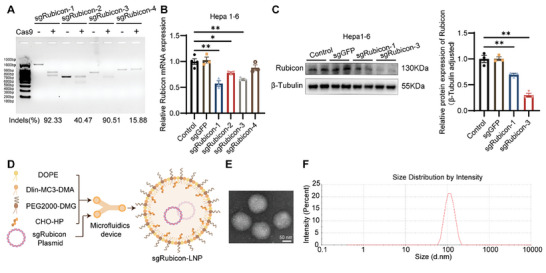
Construction and physical characterization of sgRubicon‐LNP. A) On‐target mutagenic efficiency of Rubicon sgRNAs by T7 EI assays in Hepa1‐6 cells. B) RT‐qPCR analysis of Rubicon mRNA expression levels in Hepa1‐6 cells. C) Western blot and quantitative analysis of Rubicon protein expression in Hepa1‐6 cells with indicated treatments. *β*‐Tubulin was used as a loading control (n = 3). D) Schematic illustration of sgRubicon‐LNP formulation assembled by microfluidics device. E) Representative image of sgRubicon‐LNP by TEM. Scale bars, 50 nm. F) Representative image of size distribution of sgRubicon‐LNP. The data in (B) and (C) represent means ± SEM. ^*^ *p* < 0.05, ^**^ *p* < 0.01 determined by two‐tailed *t*‐test.

To screen a highly efficient single guide RNA (sgRNA) for Rubicon knockdown, PX458 plasmids with four preliminary‐selected sgRNAs (Table S1, Supporting Information) targeting Rubicon were respectively transfected to the Hepa1‐6 cells, followed by a T7 endonuclease I (T7EI)‐based analysis of DNA mismatches in Rubicon gene sequence. As shown in **Figure** **1**A, sgRubicon‐1, and sgRubicon‐3 yielded the indel rate at 92.33% and 90.51%, respectively. Subsequent analysis revealed that sgRubicon‐3 was most efficacious in reducing Rubicon mRNA and protein levels (Figure 1B,C).

LNP was then utilized to encapsulate sgRubicon‐3 for subsequent gene delivery, which was formulated with Dlin‐MC3‐DMA, DOPE, cholesterol, and DMG‐PEG2000 by microfluidics device, named sgRubicon‐LNP (Figure 1D). The physicochemical features of sgRubicon‐LNP were characterized, revealing a homogenous spherical morphology (Figure 1E), a hydrodynamic diameter of 112 nm, polydispersity index (PDI) of 0.046, zeta potential of −0.36 mV, and entrapment efficiency over 94% (Figure 1F; Table S3, Supporting Information). Cumulatively, these results suggested that sgRubicon‐LNP was a potent delivery approach, characterized by its optimal size, homogeneity, neutral charge, and high entrapment efficiency.

### 2.2 sgRubicon‐LNP Exhibited Potent Transfection Efficiency and Reduced Liver Rubicon Expression

**Figure 2:**
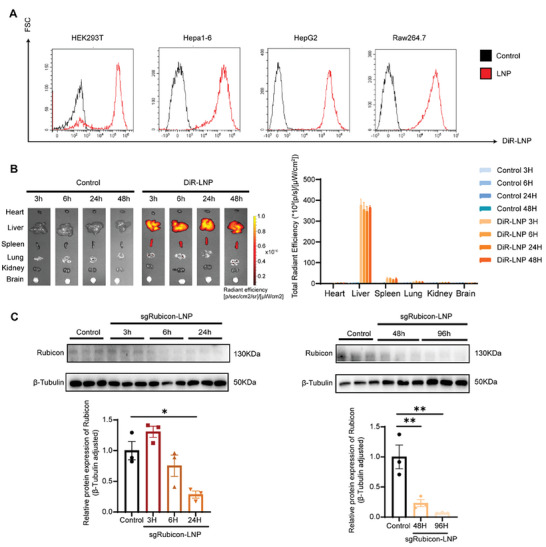
Cellular uptake, in vivo biodistribution and bioactivity assessments of sgRubicon‐LNP. A) Determination of LNP transfection capacity by flow cytometry analysis of DiR‐LNP in HEK293T, Hepa1‐6, HepG2, and Raw264.7 cells. B) IVIS representative images and quantitative analysis of DiR signal in major organs after the injection of DiR‐labelled sgRubicon‐LNP (n = 3). C) Western blot analysis of Rubicon expression in the liver of C57BL/6 mice treated with sgRubicon‐LNP. *β*‐Tubulin was used as a loading control (n = 3). The data shown in (B) and (C) were the mean ± SEM. ^*^ *p* < 0.05, ^**^ *p* < 0.01 determined by two‐tailed *t*‐test.

The cellular bio‐behavior of sgRubicon‐LNP was assessed in vitro at first. DiR Iodide (DiR) was employed for labeling sgRubicon‐LNP, followed by the LNP treated with various cells for 24 h. As shown in **Figure** **2**A, flow cytometry results showed the DiR‐positive cells were increased in HEK293T, Hepa1‐6, HepG2, and Raw264.7 cells, indicating the high cell internalization efficiency of sgRubicon‐LNP.

The in vivo performance of sgRubicon‐LNP was next examined. The administration of sgRubicon‐LNP (1 mg kg^−1^ for the plasmid) exhibited the editing efficacy of Rubicon in mice liver, which was chosen for subsequent experiments (Figure S1A, Supporting Information). Following the intravenous instruction of DiR‐labelled sgRubicon‐LNP in mice, ≈90% of the nanoparticles accumulated in the liver, while only ≈5% were detected in the spleen as quantified by the fluorescent signal (Figure 2B). Similarly, the luciferase expression was also mainly concentrated in the liver region after Luc‐LNP injection (Figure S1B, Supporting Information). The ability of sgRubicon‐LNP to silence Rubicon in mice liver was ascertained after 3, 6, 24, 48, and 96 h post‐administration of sgRubicon‐LNP, respectively. Western blot analysis revealed a time‐dependent decrease in Rubicon expression after sgRubicon‐LNP injection (Figure 2C).

Thus, it was demonstrated that sgRubicon‐LNP could mainly accumulate in livers and efficiently reduce the Rubicon expression.

### 2.3 sgRubicon‐LNP Treatment Reduced Liver Steatosis and Lipid Accumulation

**Figure 3:**
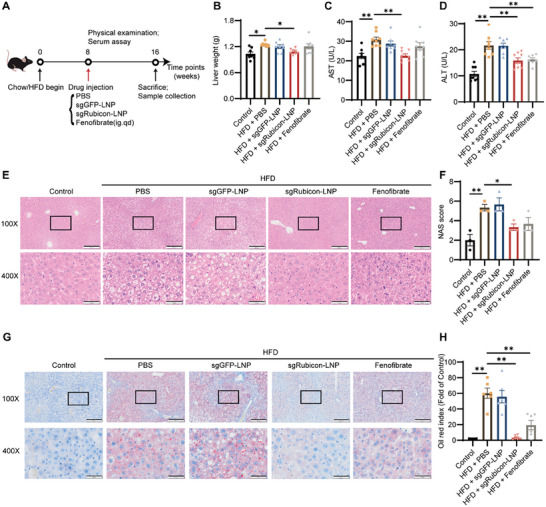
Therapeutic effects of sgRubicon‐LNP in NAFLD mice. A) Schematic illustration for the therapeutic effect evaluation of sgRubicon‐LNP against NAFLD. B) Liver weight of the mice with indicated treatments (n = 8). C,D) Quantification of serum ALT and serum AST (n  =  8). E) Representative images by H&E staining of the liver (magnification: 100 ×, 400 ×). Scale bar = 200 µm (100 ×) and 50 µm (400 ×). F) NAS score analysis (n = 3). G) Representative images by Oil Red O staining of the mice liver with indicated treatments (magnification: 100 ×, 400 ×). Scale bar = 200 µm (100 ×) and 50 µm (400 ×). H) The statistical analysis of Oil red O staining (n = 6). The data shown in (B), (C), (D), (F), and (H) were the mean ± SEM. ^*^ *p* < 0.05, ^**^ *p* < 0.01 determined by two‐tailed *t*‐test.

After being fed the high‐fat diet (HFD) for 8 weeks, mice were injected with PBS, sgGFP‐LNP, or sgRubicon‐LNP, and then observed for another 8 weeks to assess the therapeutic efficacy of sgRubicon‐LNP (**Figure** **3**A). Lower liver weight, serum aspartate aminotransferase (AST), and alanine aminotransferase (ALT) levels could be detected in mice after being treated with a single dose of sgRubicon‐LNP (Figure 3B‐D). Besides, administration of sgRubicon‐LNP also rescued histological structure in mice livers (Figure 3E,F). Oil Red O staining results exhibited the reduction of lipid deposition following sgRubicon‐LNP administration compared to the PBS or sgGFP‐LNP administration (Figure 3G,H). Meanwhile, the reduction of lipid droplets was detected in AML‐12 cells treated with Rubicon siRNA, and more lipid droplets were observed in Rubiocn overexpressed cells, indicating the alleviation of lipid accumulation after Rubicon blockage (Figure S5B,C, Supporting Information). These data indicated the ameliorating effect of sgRubicon‐LNP against liver steatosis.

**Figure 4:**
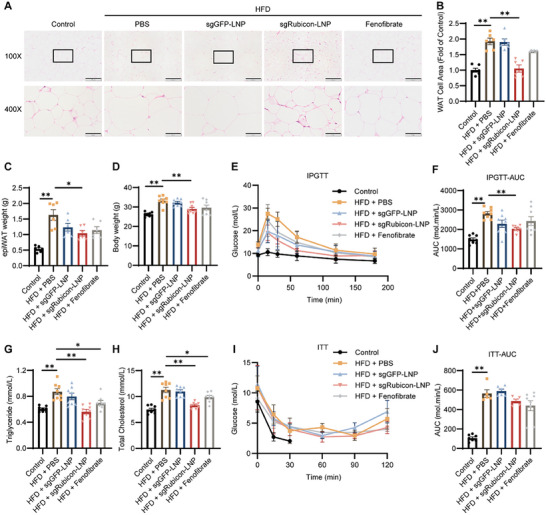
Diminished lipid accumulation and restored insulin sensitivity after sgRubicon‐LNP exposure. A) Representative images by H&E staining of the epididymal fat tissue with indicated treatments(magnification: 100 ×, 400 ×). Scale bar = 200 µm (100 X) and 50 µm (400 X). B) Analysis of relative adipose cell area (n = 6), C,D) EpiWAT weight (n = 6) and body weight of mice subjected to the indicated treatments (n = 8). E) Intraperitoneal glucose tolerance test (IPGTT, n = 8). F) Analysis of the area under curves of IPGTT (n = 8). G,H) Serum triglyceride and serum total cholesterol of mice with indicated treatments(n = 8). I) Insulin tolerance test (ITT, n = 8). J) Analysis of the area under curves of ITT (n = 5–8). The data shown in (B), (C), (D), (F), (G), (H) and (J) were the mean ± SEM. ^*^ *p* < 0.05, ^**^ *p* < 0.01 determined by two‐tailed *t*‐test.

To further confirm the regulatory effect of sgRubicon‐LNP on lipid accumulation, the size and weight of fat tissue, an important lipid storage organ, other than the liver were measured (**Figure** **4**A‐C). Remarkably, a reduction in adipocyte size, epiWAT, and body weight (Figure 4D; Figure S2A, Supporting Information) could be detected in sgRubicon‐LNP treated mice, while no significant change in food uptake was detected (Figure S2B, Supporting Information). Furthermore, elevated glucose tolerance (Figure 4E,F,I,J), along with downregulated serum triglycerides (Figure 4G) and total cholesterol (Figure 4H), could also be observed after sgRubicon‐LNP administration.

These results suggested that Rubicon knockdown alleviated hepatic steatosis and diminished lipid accumulation, likely through its regulatory effect on mice metabolism rather than reduced food uptake.

### 2.4 Rubicon‐Silencing Altered Hepatic Metabolic Profile

**Figure 5:**
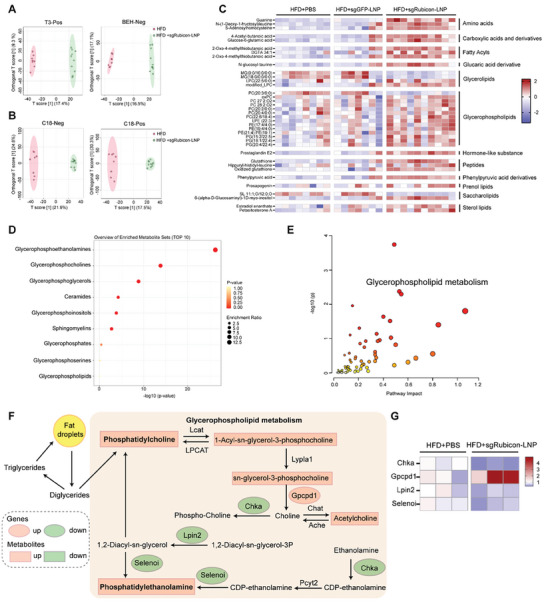
Involvement of glycerophospholipid metabolism in improved liver deposition after Rubicon blockage. A) OPLS‐DA score plots of metabolomics analysis in T3 positive and BEH negative column ionization modes using MetaboAnalyst 5.0 (n = 8–10). B) OPLS‐DA score plots of metabolomics analysis in C18 negative and positive column ionization modes (n = 8–10). C) Heat map analysis of the significantly altered metabolites among PBS, sgGFP‐LNP, and sgRubicon‐LNP treated mice. D) The enriched lipid metabolite sets interfered between PBS and sgRubicon‐LNP treated mice. E) Pathway analysis of the joint metabolomic and transcriptomic analyses. F) The biological process of glycerophospholipid metabolism. The orange and green ellipse shapes represented increased and decreased metabolites. The orange and green squares represented upregulated and downregulated genes. G) Heat map analysis of the genes implicated in the glycerophospholipid metabolism.

We then verified whether sgRubicon‐LNP could affect lipid metabolism in NAFLD mice. A metabolomics analysis using LC‐MS/MS was performed to investigate the intrahepatic metabolism profile transformation in mice liver. As shown in **Figure** **5**A and Figure S3A (Supporting Information), the Orthogonal Projections to Latent Structures Discriminant Analysis (OPLS‐DA) plots along with the volcanic maps showed that the PBS treatment and sgRubicon‐LNP treatment were independently grouped in T3 positive (T3‐Pos) mode and BEH negative (BEH‐Neg) mode (R^2^Y = 0.995, Q^2^ = 0.789 for T3‐Pos mode; R^2^Y = 0.997, Q^2^ = 0.813 for BEH‐Neg mode), indicating that the pan metabolism changed after sgRubicon‐LNP injection in NAFLD mice liver. Furthermore, the lipid metabolomics analysis was performed to explore the changes in hepatic lipid metabolism. The OPLS‐DA plots and volcanic maps revealed significant changes in C18 positive (C18‐Pos) mode and C18 negative (C18‐Neg) mode (R^2^Y = 0.99, Q^2^ = 0.658 for C18‐Pos mode; R^2^Y = 0.994, Q^2^ = 0.635 for C18‐Neg mode) (Figure 5B; Figure S3B, Supporting Information). These results suggested that sgRubicon‐LNP treatment changed the pan metabolic profile and the lipid metabolic profile in the livers of NAFLD mice.

In addition, the heatmap performed the overview of the changes of the top 50 differential metabolites (excluding those associated with LNP) in every sample. The results exhibited changes in a series of metabolites, classified as glycerophospholipids, glycerolipids, or fatty acyls, after instruction of sgRubicon‐LNP compared to PBS or sgGFP‐LNP (Figure 5C; Figure S3C, Supporting Information). Additionally, all differentially expressed metabolites derived from both C18‐Pos and C18‐Neg modes were categorized. Lipid identification results showed that glycerophosphoethanolamines, glycerophosphocholine, and glycerophosphoglycerols represented the three most abundant categories (Figure 5D), indicating the potential association between the glycerophospholipid metabolism and sgRubicon‐LNP administration.

The role of glycerophospholipid metabolism in sgRubicon‐LNP‐treated NAFLD mice was further strengthened by a joint analysis of metabolism and RNA sequencing (RNA‐seq). The RNA‐seq profiled the mRNA changes in liver after sgRubicon‐LNP injection and exhibited the enrichment in diverse lipid metabolism pathways via functional enrichment analysis (Figure S4A‐C, Supporting Information). The transcriptome and metabolome were jointly analyzed between PBS and sgRubicon‐LNP treated mice livers. Pathway analysis showed that glycerophospholipid metabolism was enriched in both differential metabolites and genes (Figure 5E). Based on the KEGG signaling pathway, as the results suggested in Figure 5F, it was probably attributed to the Rubicon‐silencing‐mediated induction of Gpcpd1 and thus activation of the phosphatidylcholine (PC) metabolism. Besides, the amount of phosphoethanolamine (PE) was increased, while the transcript level of choline kinase (*CHKa*), selenoprotein I (*Selenoi*) and Lipid phosphate phosphohydrolase II (*Lpin2*) were decreased (Figure 5G). These results indicated that Rubicon deficiency regulated hepatic lipid metabolism by activating glycerophospholipid metabolism.

### 2.5 Rubicon Silencing Reduces CD36 Expression

**Figure 6:**
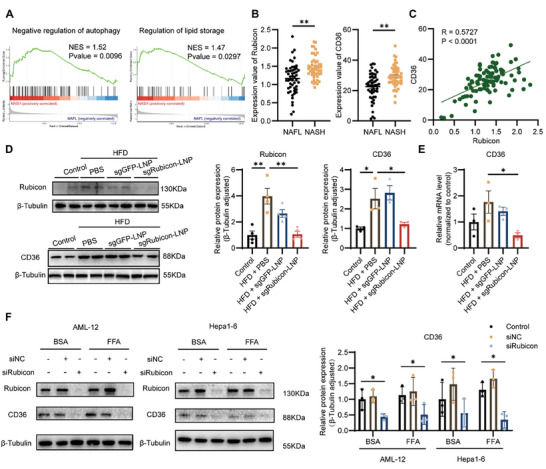
Downregulated CD36 expression by knocking down Rubicon. A) GSEA of the negative regulation of autophagy and the regulation of lipid storage in GSE167523. B) Gene expression values of Rubicon and CD36 from patients with NAFL (n = 51) and patients with NASH (n = 47). C) Correlation between Rubicon and CD36 expression values in human NAFLD patients. D) Western blot analysis of Rubicon and CD36 in the liver of mice with indicated treatment. *β*‐Tubulin was used as a loading control (n = 4). E) mRNA expression of CD36 in the liver of mice with indicated treatment. (n = 3). F) Western blot analysis of Rubicon and CD36 in AML‐12 and Hepa1‐6 cells treated with BSA or FFA (0.5 mm) for 24 h, which were transfected with siRNA against Rubicon (siRubicon) or the negative control (siNC) for 48 h. *β*‐Tubulin was used as a loading control (n = 4). The data in (B), (D), and (F) represent means ± SEM. ^*^ *p* < 0.05, ^**^ *p* < 0.01, determined by two‐tailed *t*‐test.

The specific molecular mechanism of Rubicon deficiency in lipid metabolism was investigated afterward. Given that CD36, a pathogenic factor of NAFLD, usually contributes to fatty acid uptake and triglyceride accumulation,^[^ ^22^, ^23^ ^]^ the probable relationship between CD36 and Rubicon in the NAFLD process was initially assessed. 98 patients with biopsy‐proven NAFLD, including 51 cases of NAFL samples and 47 cases of NASH samples, were collected from the Gene Expression Omnibus (GEO) database (GSE167523) (**Figure** **6**A). The Gene Set Enrichment Analysis (GSEA) revealed that the enriched pathways encompassed both the negative regulation of autophagy and the regulation of lipid storage pathways. Among the differential expression genes between NAFL and NASH patients, Rubicon was significantly upregulated (Figure 6B) and showed a positive correlation with CD36 expression (Figure 6C).

Subsequently, the modulatory effects of the Rubicon on CD36 were investigated. The expression of Rubicon along with CD36 was found to be upregulated in HFD‐fed mice, which could be decreased upon sgRubicon‐LNP administration (Figure 6D,E). The regulatory effect of Rubicon on CD36 expression was further accessed in AML‐12 and Hepa1‐6 cells. After cells were cultured with FFA and siRNA against Rubicon, CD36 expression was significantly decreased (Figure 6F; Figure S5A, Supporting Information). Collectively, these data indicated that Rubicon blocking downregulated the expression of CD36 in NAFLD.

## 3 Discussion

NAFLD is a complex metabolic syndrome primarily attributed to the dramatic lifestyle changes associated with abnormal hepatic lipid accumulation, for which therapeutic options remain limited. Rubicon, a protein with diverse physiological functions, has been identified as a critical regulator in various metabolic diseases.^[^ ^18^, ^19^, ^24^, ^25^ ^]^ Extensive research has demonstrated that Rubicon was upregulated in liver tissue of both NAFLD mice and patients. Notably, hepatocyte‐specific knockdown of Rubicon has been shown to improve liver steatosis in NAFLD mice, indicating that Rubicon may be a potential therapeutic target for NAFLD.^[^ ^20^ ^]^ In this study, a single‐dose‐usage lipid nanoparticle was developed for NAFLD treatment, designed to deliver CRISPR‐Cas9 components against Rubicon.

LNPs have shown high efficiency in nucleic acid delivery and have been approved for clinical use.^[^ ^26^ ^]^ LNPs generally contain four components: an ionizable active lipid, phosphor a helper lipid, cholesterol, and lipid‐anchored PEG. Among them, phosphor helper lipid plays a key role in the process of cellular uptake.^[^ ^27^ ^]^ Previous studies indicated that the phosphor helper lipid DSPC is suitable for delivering siRNA, a small molecule, to the liver.^[^ ^26^ ^]^ In our study, the cargo that needs to be delivered to liver is a CRISPR‐Cas9 plasmid, which is a large molecule. Therefore, DOPE, known for enhancing payload release and demonstrating high efficiency in delivering large genetic material,^[^ ^28^, ^29^ ^]^ was chosen for transporting the CRISPR‐Cas9 complex. The constructed sgRubicon‐LNP with optimal size, homogeneity, neutral charge, and high entrapment efficiency displayed high cell internalization efficiency in vitro and a significant reduction in Rubicon protein levels in vivo, demonstrating the feasibility of our strategy.

Research has shown that although MC3‐LNP accumulated in the spleen, the Fluc expression was weaker, with most expression in the liver, suggesting that accumulation in the organ may not indicate successful and efficient cellular uptake and protein expression.^[^ ^30^, ^31^ ^]^ Similarly, the sgRubicon‐LNP mainly accumulated in the liver and resulted in a significant reduction in the expression of Rubicon, while the effect of the few nanoparticles, ≈5%, observed in the spleen might be limited. Additionally, rising evidence suggested that a single administration of the CRISPR/Cas9 complex could provide stable therapeutic effects of genome editing.^[^ ^32^, ^33^, ^34^ ^]^ Qiu et al. reported that the therapeutic effect could be detected for at least 100 days after a single administration targeting ANGPTL3 in the liver.^[^ ^35^ ^]^ Our results also exhibited that a single dose of sgRubicon‐LNP could significantly reduce the expression level of Rubicon in the liver.

Current therapeutic approaches focusing on the regulation of abnormal lipid metabolism mainly include receptor agonists, therapeutic peptides, probiotics, hormones, and lipid‐lowering drugs,^[^ ^8^, ^36^ ^]^ which usually exhibit low liver selection and require short dosing intervals.^[^ ^37^, ^38^ ^]^ In contrast, our data showed that sgRubicon‐LNP had high liver selection and could retard NAFLD progression with a single administration in mice. We further investigated the therapeutic efficacy and probable mechanism of sgRubicon‐LNP in NAFLD treatment. After a single dose of the nanoparticles, the hepatic steatosis and lipid accumulation in NAFLD mice were alleviated which demonstrated that knockdown Rubicon might be a potential therapeutic strategy for NAFLD therapy.

Afterward, the impact of Rubicon knockdown on lipid metabolism regulation was investigated. The available lipidomic analysis in the livers of NAFLD patients demonstrated complex lipid signatures at different disease stages, including cholesterol, triglyceride, and phospholipids.^[^ ^39^, ^40^, ^41^ ^]^ Notably, the clinical research has shown a reduction in phospholipids, particularly PC and PE,^[^ ^42^, ^43^ ^]^ which are two major phospholipids in mammalian membranes and play a pivotal role in maintaining hepatocyte membrane functional integrity.^[^ ^44^ ^]^ The depletion of PC, considered an essential component in the NAFLD progression, caused the extracellular release of lipotoxic lipids, hepatocyte apoptosis, inflammation, and liver disease progression.^[^ ^45^ ^]^ In our study, an upregulation in the level of PC could be monitored following Rubicon blockage, indicative of the resumption of PC homeostasis and alleviation of NAFLD progression in sgRubicon‐LNP treated mice. Alterations in essential metabolic enzyme genes including *Gpcpd1*, *Chka*, *Lpin2*, and *Selenoi*, suggested the activation of the glycerophospholipid pathway and a concurrent decrease in the triglyceride synthesis pathway. Furthermore, previous research has shown an association among downregulated PE, impaired autophagy, and enhanced inflammatory response in metabolic diseases.^[^ ^20^, ^46^ ^]^ Fortunately, treatment with sgRubicon‐LNP led to an upregulation of PE level and restored autophagy activity (Figure S6, Supporting Information) in NAFLD mice. Consequently, this suggests a potential alleviating effect of Rubicon blockage on the inflammation associated with NAFLD.

Autophagy, a fundamental cellular degradation pathway, heightens the effectivity of intracellular material exchange and contributes to lipid mobilization and glycogen hydrolysis, thereby playing a pivotal role in the homeostasis of hepatic and systemic metabolism.^[^ ^47^ ^]^ The dysfunction of hepatocytic autophagy interferes with hepatic homeostasis, leading to hepatic steatosis and inflammation, which contribute to NAFLD.^[^ ^48^, ^49^, ^50^ ^]^ Recent reports investigated the potential connection of glycerophospholipid and autophagy in NAFLD and proposed some regulatory molecules.^[^ ^51^, ^52^ ^]^ Research exhibited the capabilities of nanoparticles in modulating autophagy activity for the treatment of diseases.^[^ ^53^, ^54^, ^55^ ^]^ In our results, injection of sgRubicon‐LNP reduced the expression of Rubicon, the negative regulator of autophagy, and restored autophagy activity, which exhibited amelioration of hepatic steatosis and diminished lipid accumulation.

CD36 is a key molecule that leads to signal transduction of intracellular fatty acid storage, modulating intracellular fatty acid homeostasis and the pathological process of NAFLD.^[^ ^23^, ^56^, ^57^ ^]^ Rubicon has been confirmed to participate in the lipid metabolic progress in adipocytes by regulating the adipogenic genes including CD36, indicating a strong association between Rubicon and adipogenesis.^[^ ^18^ ^]^ Our preliminary analysis of data from NAFLD patients illustrated a positive relationship between Rubicon and CD36. A significant reduction of CD36 was detected after sgRubicon blockage in both mice liver and cells. Thus, we hypothesized that Rubicon regulated CD36 expression and was likely to modulate intracellular fatty acid homeostasis, which might be related to the alleviation of lipid accumulation after Rubicon silence in NAFLD mice.

**Figure 7:**
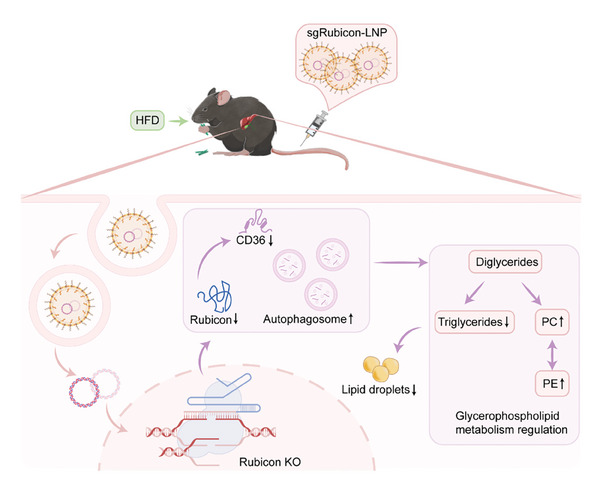
Role of sgRubicon‐LNP in the alleviated lipid accumulation in NAFLD mice.

In conclusion, we proposed a novel therapeutic approach, sgRubicon‐LNP, for NAFLD therapy via lipid nanoparticles targeting Rubicon (**Figure** **7**). A single administration of sgRubicon‐LNP reduced hepatic steatosis and lipid accumulation, regulated the glycerophospholipid metabolism pathway, and reduced CD36 expression, showcasing its potential to alleviate NAFLD. These findings highlighted the potential of Rubicon blockage as a therapeutic approach for NAFLD.

## 4 Experimental Section

### sgRNA Screening

According to the previous study,^[^ ^58^ ^]^ the efficiency of sgRNA cleavage was verified by the T7EI endonuclease assay. The Hepa1‐6 cells were harvested for direct lysis and PCR amplification of the gene surrounding the target site, followed by incubation with T7EI endonuclease (EN303, Vazyme Biotech Co.Ltd) for 30 min. The products were co‐incubated with Cas9 enzyme (D0511S, Beyotime) and analyzed on 2% agarose gels. Meanwhile, the Hepa1‐6 cells were harvested to investigate the mRNA and protein expression of Rubicon.

### LNP Formulation and Characterization

Lipid nanoparticles (LNP) were constructed according to previous literature reports.^[^ ^59^ ^]^ The lipid phase was prepared in ethanol absolute containing Dlin‐MC3‐DMA, DOPE, Cholesterol, and PEG2000‐DMG (AVT Pharmaceutical Tech Co., Ltd.) at 50:10:38.5:1.5 molar ratio. The nucleic acid phase was produced in citrate buffer (10 mm, pH 5.0) containing sgRubicon‐3 plasmid (PX458, Addgene) or luciferase plasmid (pGL4.50, Hedgehog Bio Science and Technology Ltd).

The lipid phase and nucleic acid phase were combined using a weight ratio of 10:1 (ionizable lipid: plasmid) with the final volume ratio of 1:3 by the microfluidics device (INano E, Micro&Nano Biologics Co., Ltd.). The LNPs were further filtered with 0.22 µm membrane (SLGPR33RB, Millipore) and stored at 4 °C. For the characterization, the LNPs were diluted in water at the ratio of 1:200 for size, PDI, or zeta potential measurements via Malvern Zetasizer Nano‐ZSP. The entrapment efficiency of plasmids was measured by Qubit dsDNA HS analysis kit (12640ES60, Yeasen), which quantified the amount of free plasmid, referring to previous reports.^[^ ^60^ ^]^ The representative image of LNP was visualized via 120 kV transmission electron microscopy (TEM).

### LNP Cellular Uptake and In Vivo Biodistribution

To measure the transfection capacity of LNP, the DiR‐labelled LNPs were constructed for in vitro and in vivo tracing experiments. The DiR (D4006, UElandy) was loaded to the lipid mixture at a molar ratio of 5%, according to the previous study.^[^ ^61^ ^]^ For cellular uptake analysis, DiR‐LNP (1 µg mL^−1^ of plasmid) was directly added to the culture medium of HEK293T, Hepa1‐6, HepG2, and Raw264.7 cells for 24 h, and the DiR^+^ cells were assessed by flow cytometry. For in vivo biodistribution analysis, the mice were injected intravenously with different doses of sgRubicon‐LNP (0.5, 1, or 2 mg kg^−1^ of plasmid) for 24 h and the mutagenic efficiency of Rubicon in mice liver was detected by T7EI endonuclease assay. Then the mice were treated with DiR‐labelled sgRubicon‐LNP (1 mg kg^−1^ of plasmid) and then performed the *ex vivo* organs via IVIS at 3, 6, 24, and 48 h. To quantify the Rubicon silence ability of sgRubicon‐LNP in the liver tissues, the liver samples were collected at 3, 6, 24, 48, and 96 h for western blot analysis. Meanwhile, the mice injected with the Luc‐LNP (LNP containing luciferase plasmid) were also detected by IVIS at 24 h.

### Cell Culture and Treatment

AML‐12, Hepa1‐6, HEK293T, Raw264.7, and HepG2 cells were acquired from Cell Bank (Shanghai, China) and cultured as required. The AML‐12 and Hepa1‐6 cells underwent transfection with siNC (UUCUCCGAACGUGUCACGUTT) and siRubicon (GAGCUGAUGAAGUGCAACAUGAUGAGC) (Gene Pharma, China) by Lipofectamine 3000 (Thermo Fisher Scientific) for 48 h to achieve the silence of Rubicon. Overexpression of CD36 in AML‐12 and Hepa1‐6 cells was realized by transfecting with the pcDNA3.1 vector containing the CD36 gene (Beijing Tsingke Biotech Co., Ltd.) via Lipofectamine 3000 for 48 h. Overexpression of Rubicon in AML‐12 cells was realized by transfecting with the pcDNA3.1 vector containing the Rubicon gene (Beijing Tsingke Biotech Co., Ltd.) via Lipofectamine 3000 for 48 h. To construct a cellular model of NAFLD, AML‐12, and Hepa1‐6 cells were treated in FFA(0.5 mm, OA:PA = 2:1, O7501, P9767, Sigma–Aldrich), according to the previous experiment.^[^ ^62^ ^]^

### Animal Studies

The C57BL/6 male mice (Cavens, China), from 6 to 8 weeks, weighting 22–24 g, were kept under pathogen‐free (SPF) conditions with a light/dark cycle of 12 h, according to the requirements (ethical permit 2020‐12‐SY‐ZXY‐01). Animals were maintained on a standard rodent chow diet for one week as a stage of adaptation, then given a chow diet (10010, Sino Biotechnology Siping Co. Ltd) or a high‐fat diet (HFD) (10060, Sino Biotechnology Siping Co. Ltd) for 16 weeks. The mice were intravenously treated for a single dose with PBS, sgGFP‐LNP, or sgRubicon‐LNP (1 mg kg^−1^ of plasmid), according to the previous study,^[^ ^63^ ^]^ at the 8th week or treated with Fenofibrate by intragastric administration once a day for 8 weeks.

### Biochemistry Analysis

For biochemical analysis, the alanine aminotransferase (ALT), aspartate aminotransferase (AST), total cholesterol, and triglycerides were accessed (Jiancheng Bioengineering, Nanjing). Before collecting samples, for ITT, all mice were intraperitoneally treated with insulin (1 IU kg^−1^) after 4 h (P3376, Beyotime), and then serum samples were harvested at 0, 15, 30, 60, 90, and 120 min to examine glucose. Meanwhile, all mice were injected intraperitoneally with glucose (1 g kg^−1^) after 6 h fast for IPGTT, and then serum samples were collected at 0, 15, 30, 60, 120, and 180 min to examine glucose. Food uptake was calculated as the difference in weight of food consumed over 24 h divided by the number of mice.

### Histological Analysis and Oil Red O Staining

After dissecting the mice, the liver samples and epiWAT samples were fixed by formalin, embedded with paraffin, and then treated with H&E staining. Meanwhile, formalin‐fixed liver samples embedded in OTC were subjected to Oil Red O staining. For cellular experiments, cells were cultured with 4% paraformaldehyde (G1101, Wuhan Servicebio Biotechnology Co., Ltd.), stained with working solution (S19039, Shanghai yuan ye Bio‐Technology Co., Ltd) for 15 min at 37 °C, and then washed with 60% isopropanol and PBS, which were finally observed under an inverted microscope.

### Western Blot

To obtain total proteins, tissues and cells were ground by machine were lysed (G2002, Servicebio) supplemented inhibitors (G2006, Servicebio), and then the concentration was analyzed (MA0082, MeilunBio). Total protein was resolved on SDS‐PAGE (C671102, Sangon Biotech), transferred to membranes (ISEQ00010, Sigma–Aldrich), blocked with skimmed milk (GC310001, Servicebio), incubated with anti‐Rubicon (8465, Cell Signaling Technology), anti‐CD36 (ab252922, Abcam), anti‐*β*‐Tubulin (M30109, Abmart) antibodies, and HRP‐antibodies (31430, 31460, Thermo Fisher Scientific), and finally visualized with ECL substrate (MA0186, MeilunBio).

### RT‐qPCR

Total RNA was isolated using Trizol (Vazyme Biotech Co.Ltd, R401), reverse transcribed into cDNA, and then performed by SYBR to measure the gene expression levels (Vazyme Biotech Co.Ltd, R333, Q711). The primer pairs were in Table S2 (Supporting Information).

### RNA‐seq and Data Processing

Total mRNA from liver samples was extracted. After examining the concentration, purity, and integrity, cDNA libraries were constructed, sequenced them with Illumina Novaseq 6000, and aligned to Ensembl mouse (mm10/GRCm38) reference genome. Differential expression analysis was conducted utilizing DESeq2 to identify DEGs with *P* value<0.05. Furthermore, GO and KEGG were performed by Goatools and Python Scipy.

### Bioinformatics Analysis

The data of GSE167523 was obtained from the public database GEO DataSets, which was the transcriptome profiling of human, containing 51 NAFL patients and 47 NASH patients. The differential genes and gene functional enrichment were investigated via R programming language (version 4.2.2). The GSEA analysis was plotted by https://www.bioinformatics.com.cn.

### Metabolomics Analysis and Materials

The liver samples were prepared according to previous literature reports.^[^ ^64^ ^]^ The metabolomics data of samples was collected through a combination of UHPLC (ExionLC 2.0, AB SCIEX) and mass spectrometer (X500B, AB SCIEX). The T3, BEH, C18 (176001131, 186003539, Waters; 00D‐4462‐AN, Phenomenex) was utilized for the separation of metabolites and lipids. Total 52 pairs (40 samples, 6 pooled QC, and 6 Blank) raw data files were processed using ProteoWizard to covert them into mzXML and mgf formats while centralizing them. The peaks relative quantitative matrixes were generated by our metabolomics data processing software on a large scale (2023SR0256527). Metabolite peaks were primarily annotated using the MetDNA2 (http://metdna.zhulab.cn/), while the Lipid4DAnalyzer (http://lipid4danalyzer.zhulab.cn/) was used for lipid peaks. Follow‐up analyses were performed using Metaboanalyst 5.0 (https://www.metaboanalyst.ca/).^[^ ^65^ ^]^

### Statistical Analysis

Data were expressed as mean ± SEM. Student's *t*‐test was used to compare values between the two groups via GraphPad Prism 9.0. The *P*‐value < 0.05 was considered statistically significant.

## Conflict of Interest

The authors declare no conflict of interest.

## Author Contributions

J.F. and D.J. provided guidance on research direction. Y.B., A.Z., and X.X. were responsible for writing the manuscript and designing the experimental protocol. Y.B., Y.N., and W.T. performed all experiments and data collection and analysis. Y.D. and Y.S. assisted in the metabolomics analysis. Y.B., R.Z., and Z.D. helped in lipid nanoparticle preparation and experimental assays. X.H., S.X., and Y.Z. provide technical support and corrections of the manuscript. Y.B., Y.N., and W.T. carried out grammar correction and proofreading of the article. All authors read and approved the final paper.

## Supporting information

Supporting Information

## Data Availability

The data that support the findings of this study are available from the corresponding author upon reasonable request.
