# Supplementary material for: Lipid Nanoparticle‐Mediated Delivery of CRISPR‐Cas9 Against Rubicon Ameliorates NAFLD by Modulating CD36 Along with Glycerophospholipid Metabolism
Source: Adv Sci (Weinh). 2024 Jun 18;11(31):2400493. doi: 10.1002/advs.202400493 (PMC11336963; doi:10.1002/advs.202400493)
Supplement: Supplementary file 1 — Supporting Information [file ADVS-11-2400493-s001.pdf]

ADVANCED SCIENCE
Open Access

Supporting Information

for *Adv. Sci.*, DOI 10.1002/advs.202400493

Lipid Nanoparticle-Mediated Delivery of CRISPR-Cas9 Against Rubicon Ameliorates NAFLD by Modulating CD36 Along with Glycerophospholipid Metabolism

Yu Bai, Yanyang Nan, Tao Wu, An Zhu, Xinlei Xie, Yun Sun, Yong Deng, Zihan Dou, Xiaozhi Hu, Rongrui Zhou, Shuwen Xu, Yuanzhen Zhang, Jiajun Fan and Dianwen Ju**

Supporting Information

Table. S1. sgRNA sequences for Rubicon.

Name	Sequence
sgGFP	GACCAGGATGGGCACCACCC
sgRubicon-1	GATGAGCCAGTGTCTAGAAG
sgRubicon-2	GGGTAATTTGAAGACGACTG
sgRubicon-3	CCGGAGCACCCTCAAGAGAG
sgRubicon-4	GCACTCTGACTAGCCCCCTAG

Table. S2. Primer pairs used for qPT-PCR.

Name	Sequence Forward	Sequence Reverse
β-actin	AGGTGACACATTGCTTCTG	GCTGCCTCAACACCTCAAC
Rubicon	GTCGCTCTCATGCAAACTGA	CTCATCCATGACCAGGTGTG
CD36	GCTCGTTTCAACTCTCACACAC	CATGTCTCCGACTGGCATGA

Table. S3. Table summarizing LNP physicochemical characterization.

Mean Diameter (nm)	PDI	Zeta Potential (mV)	Entrapment Efficiency (%)
112.97 ± 1.68	0.046 ± 0.018	-0.36 ± 0.93	94.47 ± 1.92

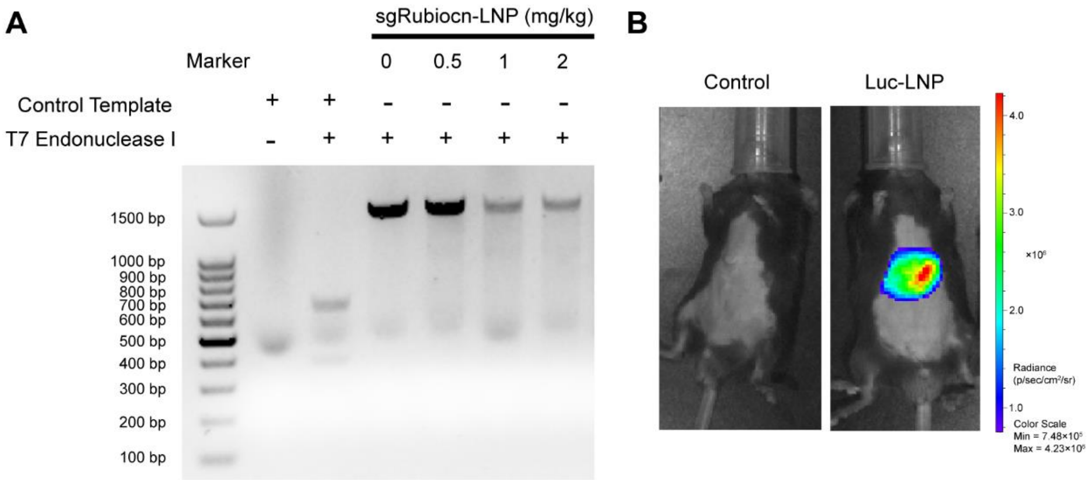

Figure S1. Dose study and biological behavior *in vivo*.
(**A**) The mutagenic efficiency of different doses of sgRubicon-LNP by T7 EI assays in mice liver. (**B**) IVIS representative images of luciferase signal in mice after the injection of Luc-LNP for 24 h.

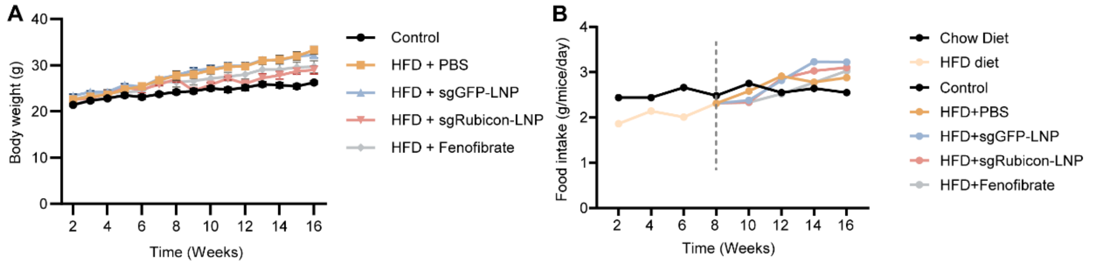

Figure S2. The body weight and food uptake.

(**A**) Body weight of each group after chow or HFD diet for 16 weeks. (**B**) The analysis of food uptake.

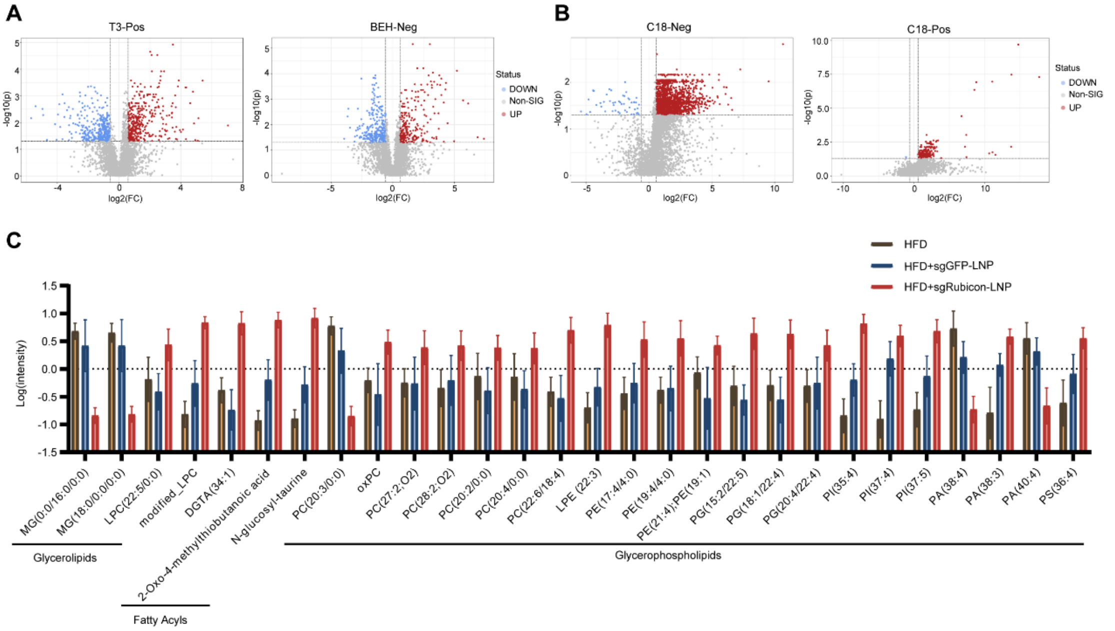

Figure S3. The metabolome-related data.

(**A**) Volcano maps displayed differential metabolites in T3 positive and BEH negative column ionization modes using MetaboAnalyst 5.0. (**B**) Volcano maps displayed differential metabolites in C18 positive and negative column ionization modes using MetaboAnalyst 5.0. (**C**) The significantly altered metabolites of glycerolipids, fatty acyls and glycerophospholipids in PBS or sgGFP-LNP or sgRubicon-LNP treated HFD-fed mice.

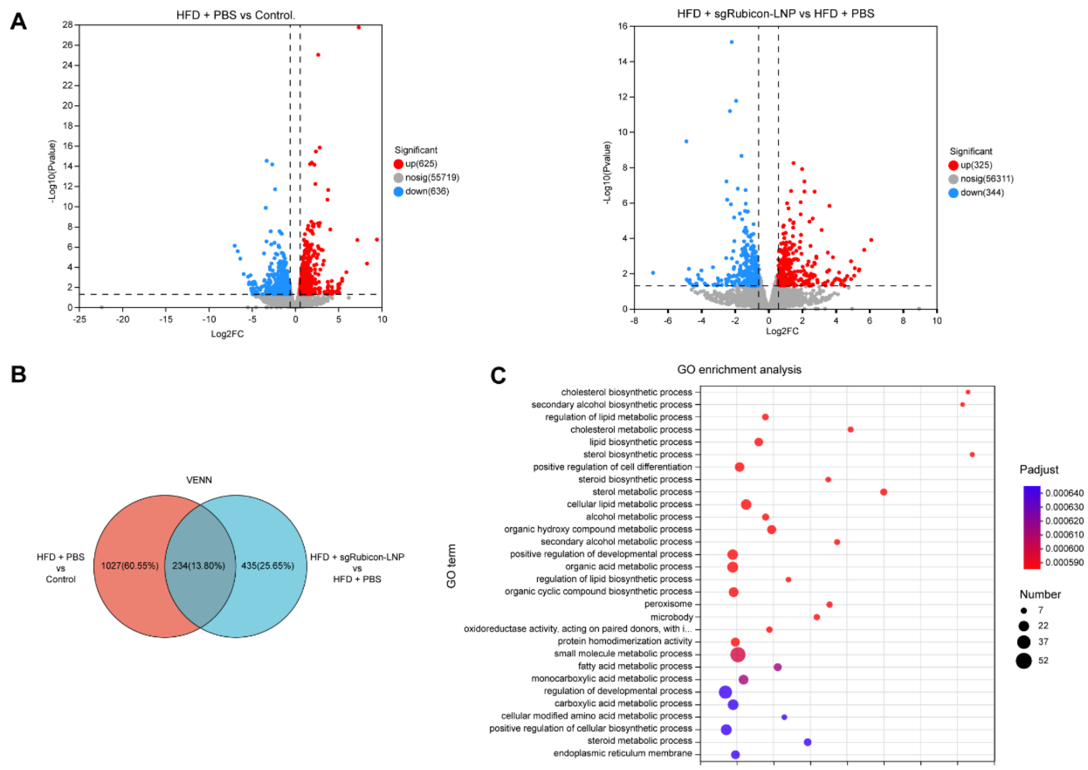

Figure S4. The RNA-seq data.

(**A**) Volcano plot for genes with differential mRNA levels between HFD-fed and control mice, sgRubicon-LNP and PBS treated HFD-fed mice. (**B**) Venn diagram analysis of gene expression among the differential genes of control vs HFD + PBS group and HFD + sgRubicon-LNP vs HFD +PBS group. (**C**) GO enrichment analysis of the common intersection genes in the Venn analysis.

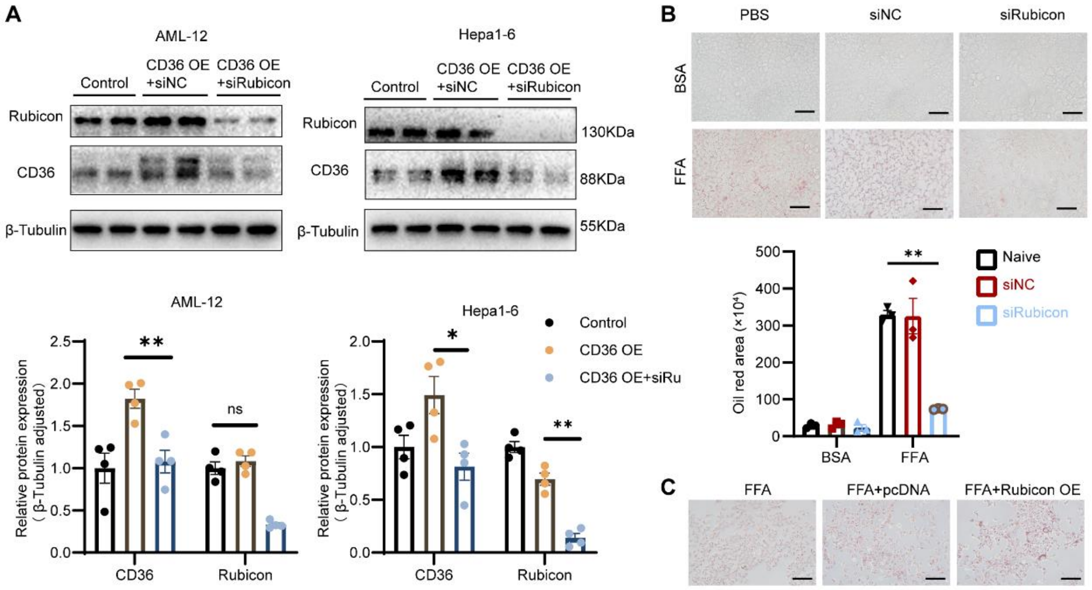

Figure S5. Western blot and the Oil red staining *in vitro*.

(**A**) Western blot analysis of Rubicon and CD36 in AML-12 and Hepa1-6 cells transfected with CD36 overexpression plasmid or siRNA for 48 hours. β-Tubulin was used as a loading

control (n = 4). (**B**) Oil Red O staining in AML-12 cells transfected with siNC or siRubicon for 48 hours and cultured with BSA or FFA for 24 hours. Scale bars, 100 μm. (**C**) Oil Red O staining in AML-12 cells transfected with pcDNA or Rubicon overexpression plasmid for 48 hours and cultured with FFA for 24 hours. Scale bars, 100 μm. The data in (**A**) and (**B**) represent means ± SEM. *P < 0.05, **P < 0.01, determined by two-tailed t-test.

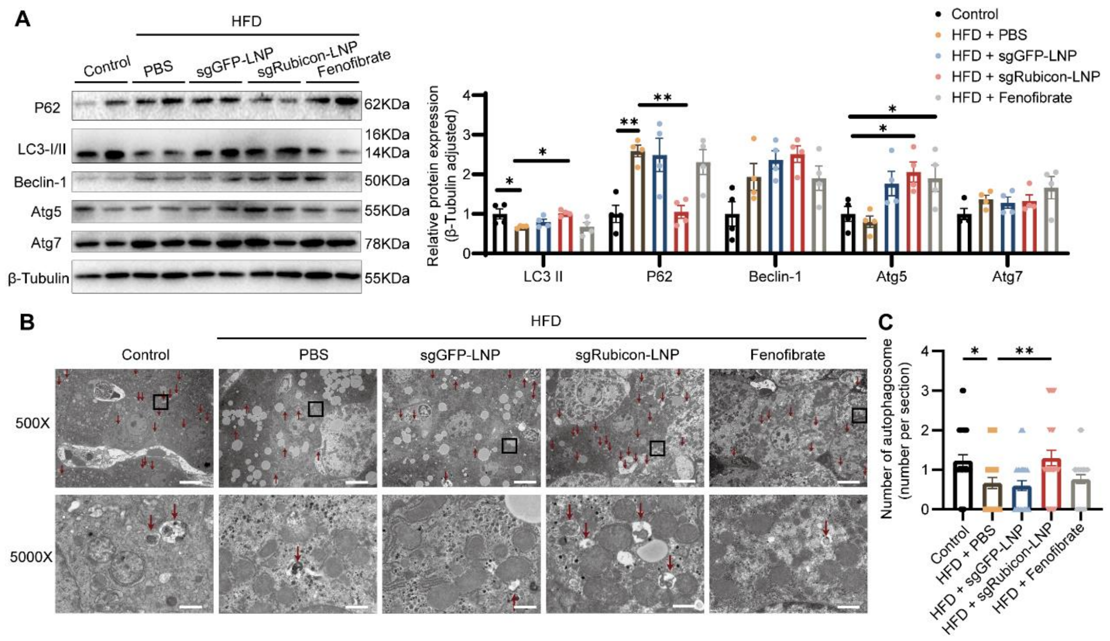

Figure S6. The improvement of autophagy by sgRubicon-LNP.

(**A**) Western blot of hepatic expression analysis for autophagic markers P62 and LC3I/II protein levels and autophagic relative protein levels such as Beclin-1, Atg5, and Atg7 in mice liver with indicated treatments (n = 4). β-Tubulin was used as a loading control. (**B**) Representative electron microscopy images of intracellular autophagic vesicles in mice liver sections with indicated treatments (magnification: 500 ×, 5000 ×;). Scale bar = 100 μm (500 X) and 10 μm (5000 X). Red arrows indicate autophagosomes. (**C**) Morphometric quantification of the number of autophagosomes in the microscopy images (n = 6). The data in (**A**) and (**C**) represent means ± SEM. *P < 0.05, **P < 0.01, determined by two-tailed t-test.
